# Lesion-specific coronary artery calcium score to predict stent underexpansion

**DOI:** 10.3389/fcvm.2025.1524390

**Published:** 2025-02-04

**Authors:** Wentao Yang, Ke Xu, Xi Fu, Weifeng Zhang, Ziyong Hao, Zhenchi Sang, Lisheng Jiang, Xingbiao Qiu, Shengxian Tu, Linghong Shen, Ben He

**Affiliations:** ^1^ Department of Cardiology, Shanghai Chest Hospital, School of Medicine, Shanghai Jiao Tong University, Shanghai, China; ^2^ Biomedical Instrument Institute, School of Biomedical Engineering, Shanghai Jiao Tong University, Shanghai, China

**Keywords:** coronary artery calcium score, percutaneous coronary intervention, coronary computed tomography angiography, non-gated non-contrast chest computed tomography, stent expansion

## Abstract

**Background:**

Previous intracoronary imaging studies have shown that coronary artery calcium (CAC) is an independent risk factor of stent underexpansion; however, limited preintervention assessments of CAC have been performed using noninvasive methods. We aimed to determine the association between lesion-specific CAC score and stent underexpansion.

**Methods:**

In this retrospective observational study, we included 416 lesions from 359 patients who underwent intravascular ultrasound (IVUS)-guided stent implantation. CAC of each lesion was quantified using the Agatston method derived from either nongated noncontrast chest CT (NCCT) or electrocardiogram-gated coronary CT angiography (CCTA). The primary endpoint was stent underexpansion defined as minimum stent area of <80% of the average reference lumen area.

**Results:**

Overall, stent underexpansion occurred in 144 (34.6%) of 416 lesions. Lesion-specific CAC score was significantly negatively correlated with the stent expansion rate (in NCCT cohort, *r* = 0.8113, *P* < 0.05; in CCTA cohort, *r* = 0.8024, *P* < 0.05). The optimal cutoff values of lesion-specific CAC score to predict stent underexpansion were >200 in both NCCT (sensitivity, 91.4%; specificity, 66.8%) and CCTA (sensitivity, 84.6%; specificity, 64.3%) cohort, which were associated with 24.94-fold increased risk of stent underexpansion in NCCT cohort and 13.56-fold increased risk of stent underexpansion in CCTA cohort.

**Conclusions:**

In this study, we found that lesion-specific CAC scores in both NCCT and CCTA cohorts were significantly independently associated with an increased risk of stent underexpansion, and the cutoff value to predict stent underexpansion was >200.

## Introduction

Stent underexpansion may lead to increased risks of the early and late complications of percutaneous coronary intervention (PCI) and stent failure (1–3). PCI of calcified lesions is associated with higher risks of adverse procedural and clinical events, often owing to suboptimal lesion modification before stent implantation and stent underexpansion due to severe coronary artery calcium (CAC). Thus, assessing the severity of calcification in coronary lesions is clinically relevant because it could help identify lesions with a higher risk of stent underexpansion and guide the application of more aggressive lesion modification strategies.

To improve the plaque modification of lesions with severe CAC and reduce the risk of stent underexpansion, several interventional techniques, including balloon technologies (e.g., scoring and cutting balloon, super high-pressure balloon, and lithoplasty balloon), atherectomy (orbital and rotational), excimer laser coronary angioplasty, and intravascular lithotripsy are currently available (4). However, how to identify the lesions with severe CAC and requiring these aggressive modification techniques has not been comprehensively investigated.

Previous studies explored the use of intracoronary imaging tools [e.g., intravascular ultrasound [IVUS] and optical coherence tomography [OCT]] in assessing CAC severity (5, 6). Fujino et al. established an OCT-based calcium scoring system (assessing three OCT parameters: maximum angle >180°, maximum thickness >0.5 mm, length >5 mm), and found that calcified lesions with a score of 4 had poor stent expansion. The IVUS-based calcium scoring system raise by Zhang et al. (assessing four IVUS features: superficial calcium angle >270° longer than 5 mm, 360° of superficial calcium, calcified nodule, vessel diameter <3.5 mm) recommended calcium modification (orbital or rotational atherectomy) in lesions with calcium score ≥2. Since these intracoronary imaging tools can improve the outcome of PCI, they are highly recommended for procedural guidance in calcified lesions (7). However, considering that intracoronary imaging catheters may not be able to cross all of the calcified lesions before modification, preintervention assessment of calcified lesions is necessary.

In contrast to intracoronary imaging tools, computed tomography (CT) is a noninvasive tool for CAC assessment, which can be performed before the procedure. CAC is conventionally quantified using images of electrocardiogram (ECG)-gated coronary CT angiography (CCTA) by the Agatston method (8), which has been widely used in predicting cardiovascular risk and guiding primary prevention of cardiovascular disease (9, 10). Besides, CAC score has predictive value in predicting obstructive coronary artery disease, and lesion-specific CAC score may be superior to the whole-heart CAC score (11). Previous studies showed that the CAC score is associated with stent expansion and might help guide the use of rotational atherectomy during PCI (12, 13). Compared with CCTA, nongated noncontrast chest CT (NCCT) is simpler and more feasible in clinical practice. NCCT had a high agreement and correlation with CCTA when assessing CAC severity (14–16). Our group previously illustrated the associations of both NCCT- and CCTA-derived lesion-specific CAC scores with increased risk of ISR (17). However, the predictive role of the lesion-specific CAC score assessed by either NCCT and CCTA in stent underexpansion remains unclear. Accordingly, we performed this study to explore the associations between CCTA- or NCCT-derived lesion-specific CAC scores and stent underexpansion.

## Materials and methods

### Study design

This was a single-center, retrospective, observational study performed at Shanghai Chest Hospital, Shanghai Jiao Tong University School of Medicine. The study protocol followed the principles of the Declaration of Helsinki and was approved by our Institutional Review Board (IS23028). The need for written informed consent was waived.

### Study population

We retrospectively screened adult patients (>18 years old) who underwent IVUS-guided drug-eluting stent (DES) implantation in *de novo* lesions in native coronary arteries at the Department of Cardiology, Shanghai Chest Hospital School of Medicine, Shanghai Jiao Tong University, from January 2020 to December 2021. Inclusion criteria were (1) lesions with calcium in CT (defined as plaque density >130 Hounsfield units with an area >1mm^2^), (2) both pre-PCI and post-PCI IVUS images with analyzable quality, and (3) analyzable CT (NCCT or CCTA) images taken within 3 months before the procedure. Exclusion criteria included (1) chronic total occlusion (CTO), (2) in-stent restenosis, (3) left main (LM) or ostial lesion, and (4) previous stenting in the target vessel. Thus, we screened 1001 lesions in 846 patients receiving IVUS-guided DES implantation, and finally included 416 lesions in 359 patients who met all of the inclusion criteria and none of the exclusion criteria in this study. According to the type of CT scan, 307 lesions were in the NCCT cohort and 109 lesions were in the CCTA cohort.

### Study endpoint

The endpoint of this study was stent underexpansion in the final post-PCI IVUS measurement, defined as a minimum stent area (MSA) of <80% of the average (proximal and distal) reference lumen area, in accordance with the European Association of Percutaneous Cardiovascular Interventions (EAPCI) expert consensus document (18).

### CT imaging acquisition and CAC scoring

Images of CCTA or NCCT were acquired using single-source ≥64-row CT scanners, and the protocols of CT scans were in accordance with the guidelines established by the Society for Cardiovascular Computed Tomography (SCCT) (19, 20). The following CT scanner systems were used: GE revolution CT, Philips iCT scanner, and Philips Ingenuity CT.

To quantify coronary calcium, we used the semiautomated software OsiriX (Pixmeo, Geneva, Switzerland) to obtain the Agatston score in noncontrast-enhanced data sets. In three main epicardial coronary arteries, namely, left anterior descending artery (LAD), left circumflex artery (LCX), and right coronary artery (RCA), we located the target lesion by using some conspicuous and reproducible anatomical landmarks such as the ostium of the main vessel and side branches (Supplementary Figure 1). As the slice thickness may differ from one patient to the next, we adjusted the Agatston score using the following formula: Adjusted score = original score × slice thickness/3 (21).

### IVUS imaging acquisition and analysis

The IVUS testing and measurement protocol followed the requirements established by the American College of Cardiology (ACC) clinical expert consensus document (22). We used commercially available mechanical IVUS catheters (OptiCross 40 MHz; BostonScientific, Marlborough, MA, USA) to obtain the images. The IVUS catheter was inserted at least 10 mm distal to the lesion and automatically pulled back to the aorta at a rate of 0.5 mm/s or 1.0 mm/s after the intracoronary administration of 100–200 μg of nitroglycerin. We initially performed an IVUS examination before the lesion preparation. If the IVUS catheter could not be delivered across the lesion, dilation using a small balloon (diameter ≤2 mm) would be conducted and IVUS catheter delivery would be re-performed.

All IVUS images were analyzed using Echoplaque software (INDEC systems, Inc., Los Altos, CA, USA) offline by two independent experienced technicians. Several definitions or criteria were used in the IVUS analysis: (1) Reference lumen was defined as the site with the largest lumen area proximal or distal to the target lesion or stent edge, but within the same segment (within 10 mm of the lesion or within 5 mm of the stent edge, and with no major intervening branches). (2) The lesion was defined as the site with obvious atherosclerotic plaque compared with a predefined reference and usually had stenosis in at least 50% of the area. (3) Calcified plaque was defined as bright echoes and obstructed the penetration of ultrasound. In pre-PCI IVUS analysis, we measured lesion length, minimum lumen area (MLA), average reference lumen diameter, maximum calcium angle, and calcium length. Besides, we also calculate the IVUS-based calcium score using Zhang's method (5). In post-PCI IVUS analysis, we measured MSA, stent area at maximum calcium, average reference lumen area, and stent expansion rate (MSA/average reference lumen area).

### Statistical analysis

We performed per-patient analyses for patient clinical characteristics, and per-lesion analyses for lesions or procedural characteristics. Continuous variables are presented as the mean ± standard deviation or median and first and third quartiles (Q1, Q3), where appropriate. Categorical variables are shown as number (percentage). We divided the lesions into high and low CAC score groups in both the NCCT cohort and the CCTA cohort according to the median CAC score. Variables were compared between the high and low CAC score groups using the Mann–Whitney test or chi-squared test, where appropriate. As an additional validation, we explored the distribution of IVUS-based calcium score due to high and low CAC score. Spearman's correlation analyses were used to assess the correlation between the CAC score and stent expansion rate. We also displayed the stent expansion rate of each lesion in both high and low CAC score groups. Receiver-operating characteristic (ROC) curve analysis was used to determine the ability of the lesion CAC score to predict stent underexpansion and identify the optimal cutoff value of the CAC score that provided the greatest sum of sensitivity and specificity in the NCCT cohort and CCTA cohort. The odds ratio (OR) and 95% confidence interval (CI) of the risk of stent underexpansion associated with the cutoff CAC score were obtained using a univariable logistic regression model. We also performed multivariable logistic regression analyses, which included the patient clinical characteristics (age, sex, smoking status, hypertension, dyslipidemia, diabetes, chronic kidney disease), procedure characteristics (total stent length, maximum inflation pressure, and device-to-artery ratio), and CAC score.

All analyses were conducted with SPSS, version 26.0 (SPSS Inc., IBM Corp., Armonk, NY, USA), and GraphPad Prism, version 9.5.0 (GraphPad Software, San Diego, CA, USA). A two-sided *P*-value of <0.05 was considered statistically significant.

## Results

### Patient clinical characteristics

**Figure 1:**
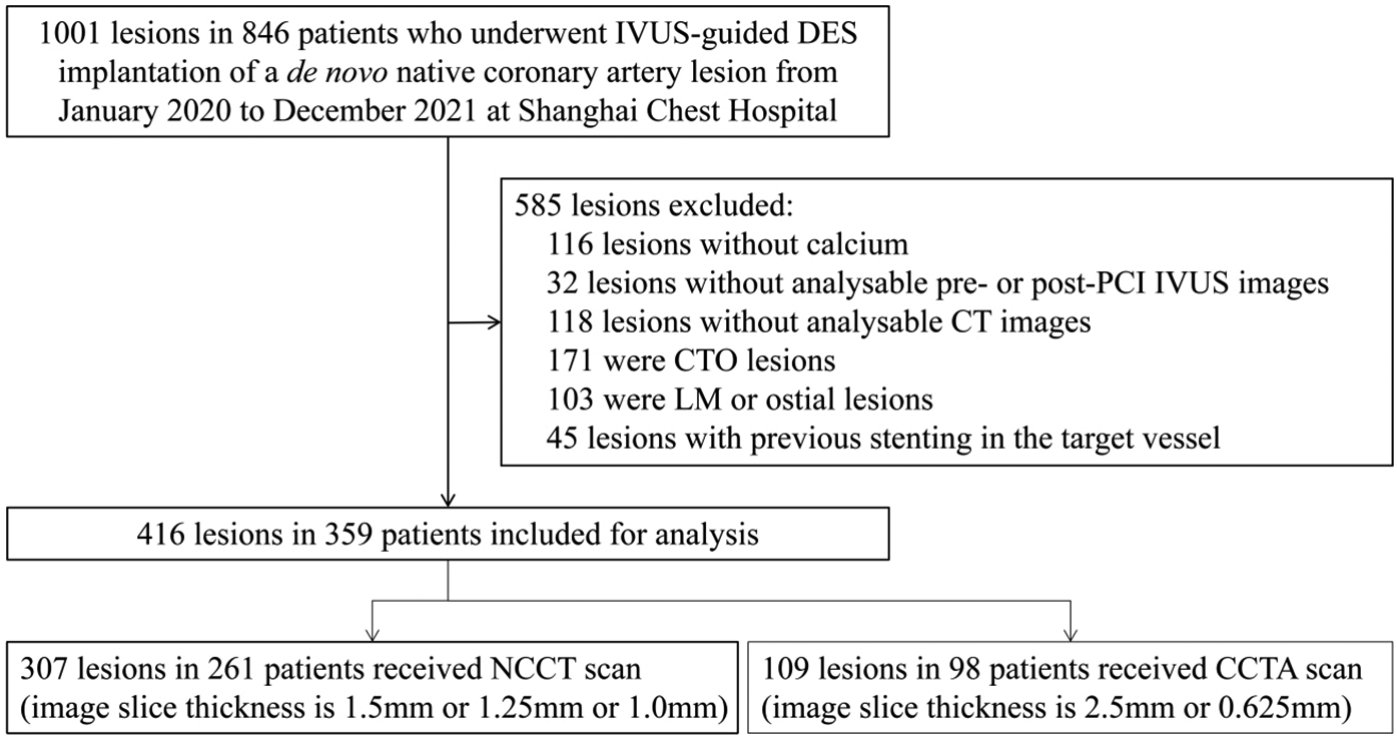
Study flow. IVUS, intravascular ultrasound; DES, drug-eluting stent; CTO, chronic total occlusion; PCI, percutaneous coronary intervention; CT, computed tomography; LM, left main; NCCT, non-gated non-contrast chest computed tomography; CCTA, coronary computed tomographic angiography.

We screened 1,001 lesions in 846 patients who underwent IVUS-guided DES implantation of a *de novo* native coronary artery lesion. Finally, 416 lesions in 359 patients who met the inclusion criteria and had none of the exclusion criteria were included in the analysis (Figure 1). The clinical characteristics of the patients in each cohort are summarized in Table 1. The mean age of the patients was 68.1 ± 7.2 years, and 69.9% were male.

**Table 1:** Patient clinical characteristics.

	Total (*n* = 359 patients)	NCCT Cohort (*n* = 261 patients)	CCTA Cohort (*n* = 98 patients)
Age (years)	68.1 ± 7.2	68.8 ± 7.5	66.3 ± 6.1
Male, *n* (%)	251 (69.9)	188 (72.0)	63 (64.3)
Smoking, *n* (%)	122 (34.0)	84 (32.2)	38 (38.8)
Hypertension, *n* (%)	271 (75.5)	197 (75.5)	74 (75.5)
Dyslipidemia, *n* (%)	251 (69.9)	179 (68.6)	72 (73.5)
Diabetes mellitus, *n* (%)	138 (38.4)	106 (40.6)	32 (32.6)
CKD, *n* (%)	37 (10.3)	28 (10.7)	9 (9.2)
Prior MI, *n* (%)	30 (8.4)	23 (9.7)	7 (7.1)
Prior PCI, *n* (%)	54 (15.0)	43 (16.5)	11 (11.2)
Diagnosis			
Stable angina, *n* (%)	229 (63.8)	169 (64.8)	60 (61.2)
Unstable angina, *n* (%)	71 (19.8)	53 (20.3)	18 (18.4)
NSTEMI, *n* (%)	59 (16.4)	39 (14.9)	20 (20.4)

Values are presented as mean ± standard deviation or *n* (%). NCCT, non-gated non-contrast chest computed tomography; CCTA, coronary computed tomographic angiography; CKD, chronic kidney disease; MI, myocardial infarction; PCI, percutaneous coronary intervention; NSTEMI, non-ST segment elevation myocardial infarction.

### Lesion and procedure characteristics

As the median lesion-specific CAC score was 218 (96, 399) in the NCCT cohort and 207 (120, 360) in the CCTA cohort, we divided the lesions into high CAC score and low CAC score groups. Table 2 shows the lesion and procedure characteristics. In the pre-PCI IVUS assessment, the high CAC score group had greater calcium length, maximum calcium angle, and IVUS-based calcium score than the low CAC score group in both cohort. In the post-PCI IVUS assessment, compared with the high CAC score group, the low CAC score group had greater MSA and rate of stent expansion at the MSA site. Other variables were comparable between the two groups. The distribution of IVUS-based calcium score according to high and low CAC score was shown in Supplementary Figure 2.

**Table 2:** Lesion and procedure characteristics.

	NCCT cohort (*n* = 307 lesions)			CCTA cohort (*n* = 109 lesions)		
	High CAC score (*n* = 153)	Low CAC score (*n* = 154)	*P* value	High CAC score (*n* = 54)	Low CAC score (*n* = 55)	*P* value
Lesion location			0.61			0.79
LAD	79 (51.6)	73 (47.4)		24 (44.4)	28 (50.9)	
LCX	34 (22.2)	33 (21.4)		13 (24.1)	12 (21.8)	
RCA	40 (26.1)	48 (31.2)		17 (31.5)	15 (27.3)	
Lesion length, mm	24.8 (18.5, 30.1)	23.9 (17.2, 27.5)	0.10	25.2 (17.4, 31.6)	24.7 (16.9, 29.7)	0.55
Reference lumen diameter, mm	2.9 (2.6, 3.3)	3.0 (2.7, 3.3)	0.45	2.8 (2.6, 3.3)	2.8 (2.6, 3.2)	0.63
Calcium length, mm	13.2 (10.8, 17.0)	12.0 (9.9, 15.4)	<0.05	13.6 (10.8, 17.4)	12.0 (9.8, 14.2)	0.06
Maximum calcium angle,°	288 (191, 360)	181 (119, 285)	<0.05	233 (173, 302)	178 (137, 276)	<0.05
IVUS-based calcium score	2 (1, 3)	1 (1, 2)	<0.05	2 (1, 3)	1 (1, 2)	<0.05
Minimum lumen area, mm^2^	2.9 (2.3, 3.4)	3.0 (2.4, 3.4)	0.18	2.7 (2.3, 3.3)	2.8 (2.3, 3.5)	0.52
MSA, mm^2^	5.2 (4.5, 6.2)	6.0 (5.4, 6.7)	<0.05	5.4 (4.2, 5.8)	6.1 (5.1, 7.1)	<0.05
Average reference lumen area, mm^2^	7.1 (5.7, 8.4)	6.7 (6.0, 7.6)	0.17	6.6 (5.7, 7.4)	6.8 (5.9, 7.4)	0.75
Stent expansion rate at MSA, %	76.8 (72.2, 81.6)	91.5 (84.8, 94.2)	<0.05	77.4 (70.7, 85.3)	92.4 (83.8, 95.9)	<0.05
Total stent length, mm	29 (22, 36)	30 (20, 38)	0.47	26 (18, 38)	26 (20, 38)	0.82
Total number of stents used	1 (1, 2)	1 (1, 2)	0.16	1 (1, 2)	1 (1, 2)	0.68
Maximum inflation pressure, atm	18 (16, 20)	18 (16, 20)	0.88	18 (16, 20)	18 (16, 20)	0.71
Scoring balloon	51 (33.1)	37 (23.9)	0.07	18 (33.3)	11 (21.8)	0.47
Cutting balloon	37 (24.2)	27 (17.5)	0.15	8 (14.8)	3 (0.05)	0.19
ELCA	4 (0.03)	0 (0.00)	0.13	1 (0.02)	0 (0.00)	0.99
RA	5 (0.03)	1 (0.01)	0.12	2 (0.04)	0 (0.00)	0.47
Device to artery ratio	1.00 (0.88, 1.07)	0.96 (0.86, 1.05)	0.19	1.00 (0.88, 1.08)	0.98 (0.89, 1.06)	0.80

Note: Values are presented as *n* (%) or median and first and third quartiles (Q1, Q3). *P* values were calculated using Mann–Whitney test or chi-squared test as appropriate. NCCT, non-gated non-contrast chest computed tomography; CCTA, coronary computed tomographic angiography; CAC, coronary artery calcium; LAD, left anterior descending artery; LCX, left circumflex artery; RCA, right coronary artery; IVUS, intravascular ultrasound; MSA, minimal stent area; ELCA, excimer-laser coronary angioplasty; RA, rotational atherectomy.

### Distribution of stent expansion rate in low and high CAC score groups

**Figure 2:**
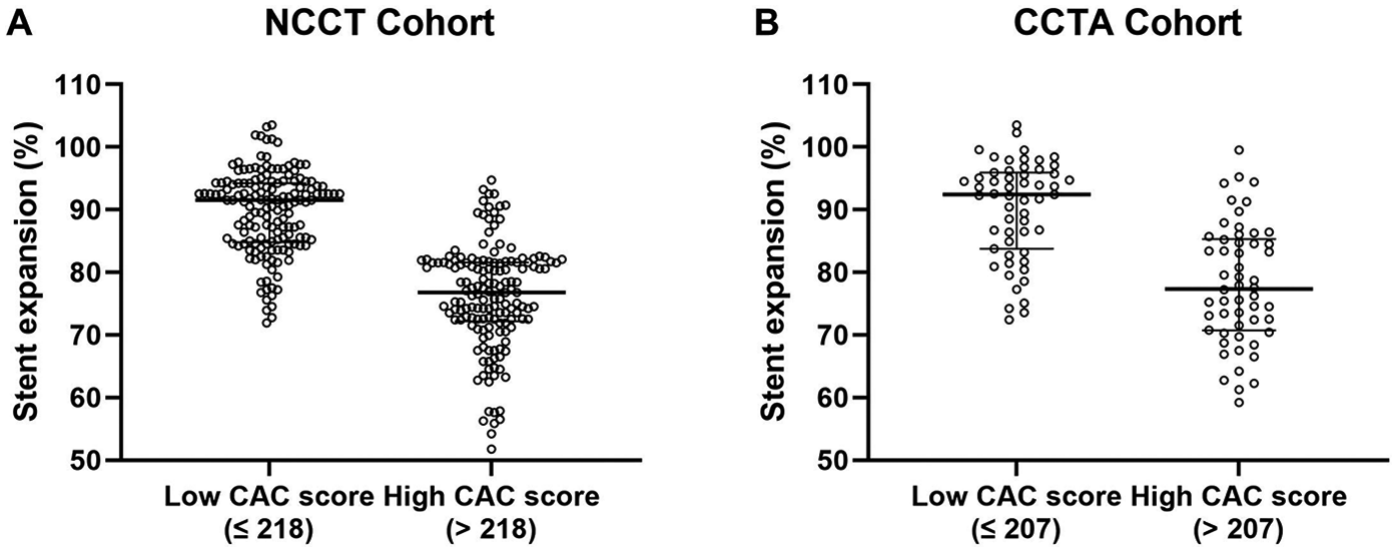
Distribution of stent expansion rate in the low and high CAC score groups in NCCT **(A)** and CCTA **(B)** cohort. NCCT, non-gated non-contrast chest computed tomography; CCTA, coronary computed tomographic angiography; CAC, coronary artery calcium.

Stent underexpansion occurred in 144 (34.6%) of 416 lesions in the whole cohort. In addition, the distribution of stent expansion rate in both cohorts according to the CAC score is presented in Figure 2. In the NCCT cohort, the median stent expansion rate was 76.8% in the high CAC score group and 91.5% in the low CAC score group; the occurrence of stent underexpansion was 60.1% in the high CAC score group and 8.4% in the low CAC score group. In the CCTA cohort, the median stent expansion rate was 77.4% in the high CAC score group and 92.4% in the low CAC score group; the occurrence of stent underexpansion was 59.2% in the high CAC score group and 12.7% in the low CAC score group.

### Correlations between the CAC score and stent expansion rate

**Figure 3:**
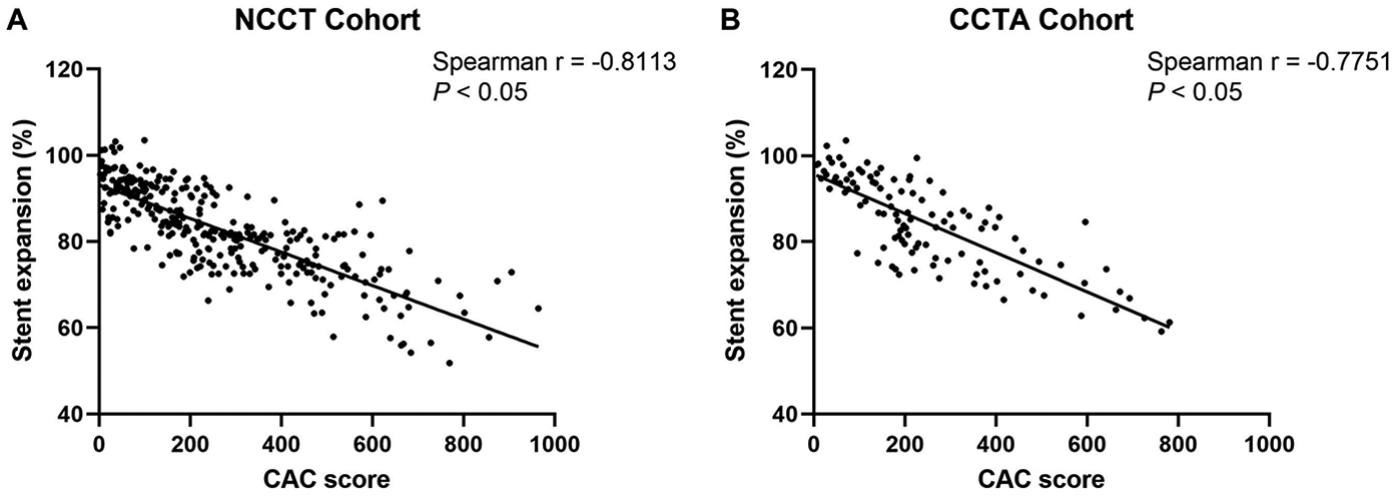
Correlations between the CAC score and stent expansion rate in NCCT **(A)** and CCTA **(B)** cohort. CAC, coronary artery calcium; NCCT, non-gated non-contrast chest computed tomography; CCTA, coronary computed tomographic angiography.

As shown in Figure 3, using Spearman's correlation analysis, the lesion-specific CAC score was significantly negatively correlated with the stent expansion rate in both cohorts (Spearman's r = −0.8113, *P* < 0.05, in NCCT cohort; Spearman's *r* = −0.8024, *P* < 0.05, in CCTA cohort).

### Receiver-operating characteristic analyses

**Figure 4:**
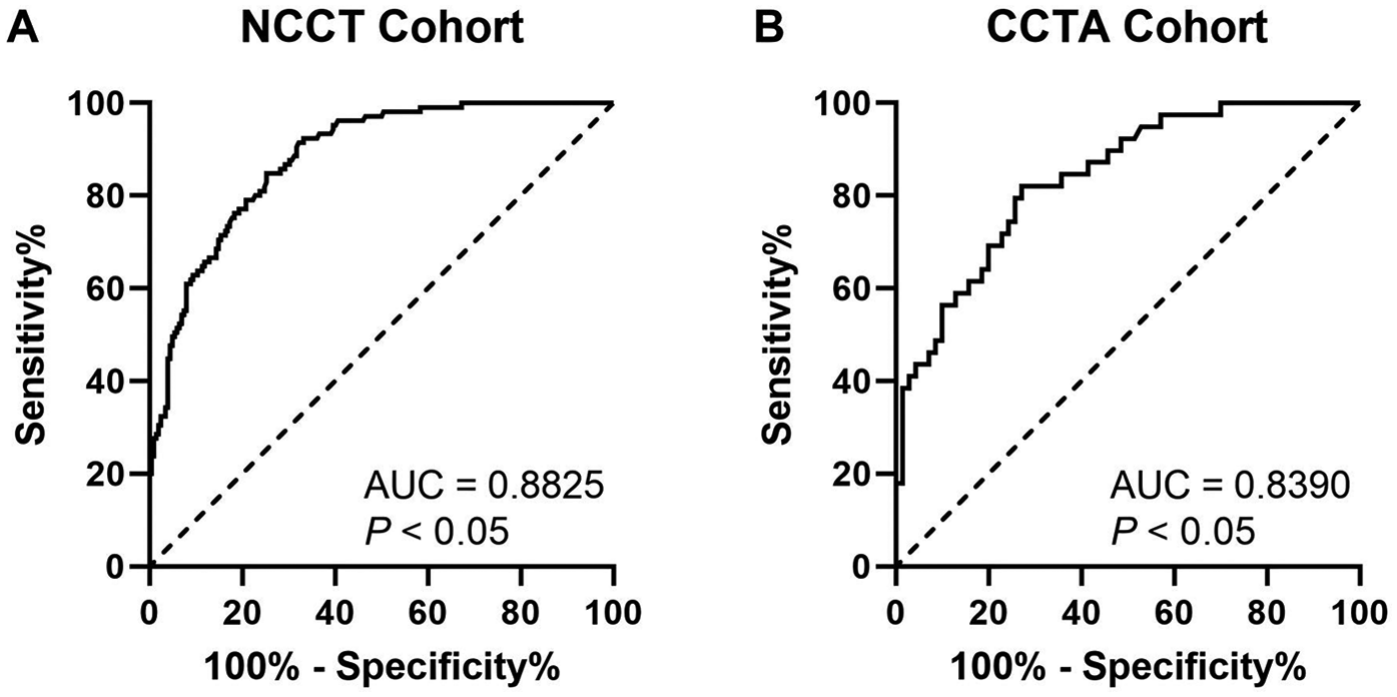
ROC curves for CAC score to predict stent underexpansion in NCCT **(A)** and CCTA **(B)** cohort. ROC, receiver operating characteristic; NCCT, non-gated non-contrast chest computed tomography; CCTA, coronary computed tomographic angiography; AUC, area under the curve.

**Figure 5:**
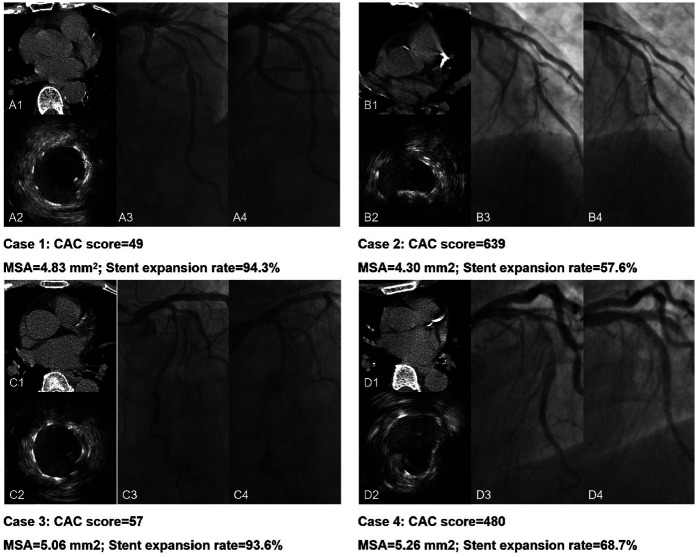
Representative case examples. Case 1. CAC score < 200 in NCCT (A1) and excellent stent expansion (A2); A3 and A4 present the pre- and post-PCI angiography of the LAD lesion. Case 2. CAC score > 200 in NCCT (B1) with stent underexpansion (B2); B3 and B4 present the pre- and post-PCI angiography of the LAD lesion. Case 3. CAC score < 200 in CCTA (C1) and excellent stent expansion (C2); C3 and C4 present the pre- and post-PCI angiography of the LAD lesion. Case 4. CAC score > 200 in CCTA (D1) with stent underexpansion (D2); D3 and D4 present the pre- and post-PCI angiography of the LAD lesion. NCCT, non-gated non-contrast chest computed tomography; CCTA, coronary computed tomographic angiography; PCI, percutaneous coronary intervention; LAD, left anterior descending artery.

In Figure 4, ROC analyses demonstrated lesion-specific CAC score to be highly sensitive and specific for predicting stent underexpansion, as indicated by an area under the curve (AUC) of 0.8825 (95% CI, 0.8457–0.9193; *P* < 0.05) in NCCT cohort and by an AUC of 0.8390 (95% CI, 0.7643–0.9137; *P* < 0.05) in CCTA cohort. Using Youden's index, we found the optimal cutoff value for predicting stent underexpansion was 200, which provided a sensitivity of 91.4% (95% CI, 84.5%–95.4%) and specificity of 66.8% (95% CI, 60.1%–73.0%) in NCCT cohort and provided a sensitivity of 84.6% (95% CI, 70.3%–92.8%) and specificity of 64.3% (95% CI, 52.6%–74.5%) in CCTA cohort. Representative case examples are shown in Figure 5.

### Univariable and multivariable logistic regression analyses

Table 3 shows the results of the univariable and multivariable logistic regression models for factors that correlated with stent underexpansion. In the univariable logistic regression model, lesion-specific CAC score >200 was associated with 21.49-fold (OR, 21.49; 95% CI, 10.22–45.20; *P* < 0.05) increased risk of stent underexpansion in the NCCT cohort and was associated with 7.74-fold (OR, 7.74; 95% CI, 2.99–20.02; *P* < 0.05) increased risk of stent underexpansion in the CCTA cohort. In the multivariable logistic regression model, after adjusting for patient characteristics (age, sex, smoking status, hypertension, dyslipidemia, diabetes, chronic kidney disease) and procedure characteristics (total stent length, maximum inflation pressure, and device-to-artery ratio), CAC score >200 was associated with 24.94-fold (OR, 24.94; 95% CI, 11.53–53.95; *P* < 0.05) increased risk of stent underexpansion in the NCCT cohort and was associated with 13.56-fold (OR, 13.56; 95% CI, 4.23–43.47; *P* < 0.05) increased risk of stent underexpansion in the CCTA cohort.

**Table 3:** Univariable and multivariable logistic regression analyses for stent underexpansion.

(A) NCCT cohort						
	Univariable analysis			Multivariable analysis		
	OR	95% CI	*P* value	OR	95% CI	*P* value
Age	1.02	0.98–1.05	0.31			
Male	0.96	0.57–1.62	0.88			
Smoking	0.90	0.54–1.50	0.70			
Hypertension	1.38	0.78–2.42	0.27			
Dyslipidemia	1.06	0.64–1.76	0.83			
Diabetes mellitus	1.21	0.75–1.95	0.43			
CKD	1.17	0.55–2.51	0.68			
Stent length	1.01	0.99–1.04	0.32			
Maximum inflation pressure	0.96	0.89–1.04	0.35			
Device to artery ratio	0.79	0.08–7.38	0.84			
Lesion-specific CAC score >200	21.49	10.22–45.20	<0.05	24.94	11.53–53.95	<0.05

(B) CCTA cohort						
	Univariable analysis			Multivariable analysis		
	OR	95% CI	*P* value	OR	95% CI	*P* value
Age	1.02	0.95–1.09	0.61			
Male	1.18	0.52–2.69	0.69			
Smoking	0.89	0.40–2.01	0.78			
Hypertension	1.15	0.46–2.89	0.76			
Dyslipidemia	1.55	0.61–3.95	0.36			
Diabetes mellitus	1.74	0.76–3.96	0.19			
CKD	1.49	0.38–5.89	0.57			
Stent length	1.00	0.97–1.05	0.69			
Maximum inflation pressure	0.95	0.82–1.10	0.49			
device to artery ratio	0.15	0.00–8.20	0.35			
Lesion-specific CAC score >200	7.74	2.99–20.02	<0.05	13.56	4.23–43.47	<0.05

NCCT, non-gated non-contrast chest computed tomography; CCTA, coronary computed tomographic angiography; CKD, chronic kidney disease; CAC, coronary artery calcium.

## Discussion

In this study, we found that lesion-specific CAC score was significantly associated with the stent expansion rate. In NCCT cohort, lesion-specific CAC score >200 was significantly associated with a 24.94-fold increased risk of stent underexpansion. In CCTA cohort, lesion-specific CAC score >200 was significantly associated with a 13.56-fold increased risk of stent underexpansion.

Previous studies have shown that stent underexpansion increases the risk of stent thrombosis and in-stent restenosis, leading to myocardial infarction, unplanned revascularization, and even death (2, 23). To reduce stent and clinical failure, good stent expansion should be achieved during PCI. The EAPCI expert consensus document of intracoronary imaging has summarized the optimization targets for stent expansion in non-LM lesions: (1) MSA >5.5 mm^2^ in IVUS or MSA >4.5 mm^2^ in OCT; and (2) MSA/average reference lumen >80% as optimal stent expansion (18). However, the incidence of stent underexpansion in severely calcified lesions is higher, resulting in higher risks of procedure and clinical failure.

With regard to the procedure, the most important factor to prevent calcium-related stent underexpansion is accurate assessment and adequate lesion preparation. Hence, an interventional cardiologist should use certain tools to identify calcified lesions at high risk of stent underexpansion, and prepare to perform atherectomy (rotational or orbital), intravascular lithotripsy, or excimer laser coronary angioplasty (4). Several imaging tools are available to evaluate coronary calcium, of which the most commonly used is coronary angiography. However, owing to the device resolution, overlapping anatomical structures, and poor delineation of nonphosphate calcium, coronary angiography is unable to accurately reveal calcified lesions. In contrast, intracoronary imaging including IVUS and OCT has higher sensitivity and specificity in demonstrating calcified lesions and correlates with the pathological findings (24, 25). When describing a calcified lesion in IVUS or OCT, several characteristics should be included: calcium length, calcium angle, calcium thickness (not in IVUS given the poor penetrability), calcium location (deep or superficial), and calcium morphology. Deep calcium is generally not considered to affect stent implantation. It is also clear that calcium morphology markedly impacts cardiovascular events and stent expansion. Calcified nodules (CNs) have been demonstrated as a cause of acute coronary syndrome (ACS) and are very common in heavily calcified lesions requiring rotational atherectomy (26, 27). Meanwhile, whether calcium length and angle can increase the risk of stent underexpansion independently is debatable, as studies have shown opposite results in both IVUS and OCT (5, 6, 28, 29).

Both coronary angiography and intracoronary imaging can only be performed in a catheter laboratory, while CT scans can provide qualitative and quantitative information about CAC noninvasively before the procedure. CAC has historically been used for the auxiliary diagnosis of coronary artery disease and cardiovascular risk stratification. Mechanistically, the CAC score is a quantitative calcium-volume evaluation tool obtained from non-contrast-enhanced data sets of CCTA scans (8). Until now, the primary use of CAC score recommended by professional societies is predicting the risk of atherosclerotic cardiovascular disease and then guiding primary prevention. There are only a few researches explore the association between CAC score and procedures or outcomes of PCI, and the utility of CAC score in PCI is far from being discovered.

When talking about CAC score derived from CT, three parameters should be distinguished: the total CAC score, the vessel CAC score and the lesion CAC score. Although the total CAC score is a reliable imaging surrogate of atherosclerosis burden, the vessel CAC score and lesion CAC score are more related to specific calcified lesion features. Qian et al. have found that vessel- and lesion-specific CAC score are superior to the whole-heart CAC score in predicting obstructive coronary artery disease (11). A recent study by Sugiura et al. had investigated the ability of CCTA in predicting CNs detected by OCT and higher CAC score of the target vessel in the CN group than the non-CN group, CACS ≥162 was the best cutoff values for predicting CNs (30). Komaki et al. have explored the association between target vessel CAC score derived from CCTA and stent expansion in PCI, they found that stent expansion rate was associated with the target vessel CAC score and the target vessel CAC score was a better predictor of stent expansion than the total CAC score (12). Sekimoto et al. have conduct a research focused on per-lesion CAC score, they found per-lesion calcium score ≥453 predicted rotablation and high per-lesion calcium score was an independent predictor of rotablation (13). At present, the lesion CAC score is more difficult to measure automatically than the total and vessel CAC score and its importance in PCI practice is not well understood. Unlike Sekimoto's study, we measured CAC score of the target lesion from both CCTA and NCCT cohort in this research and demonstrated the lesion-specific CAC score were associated with stent underexpansion.

Considering the limitations of CCTA and the technical feasibility of NCCT in routine clinical practice, CAC evaluation by NCCT has been explored by many studies. Although NCCT cannot be practically used for formal Agatston scoring, studies have shown that gated and nongated Agatston scores are highly correlated (14–16). Furthermore, the SCCT guidelines have recommended that the presence, severity, and extent of CAC be reported on noncontrast noncardiac chest CT scans (20). In our previous research, lesion-specific CAC scores in both NCCT and CCTA were significantly associated with an increased risk of in-stent restenosis, which could be a result of stent underexpansion (17). Therefore, in the current study, we explored the association between lesion-specific CAC score and stent expansion. Besides we also provided a cutoff value to predict stent underexpansion for clinical use in both CCTA and NCCT scans. When preintervention assessments reveal lesion-specific CAC score >200 in either NCCT or CCTA, the operators can more strongly consider aggressive calcium modification, such as atherectomy (rotational or orbital), intravascular lithotripsy, or excimer laser coronary angioplasty, which were mentioned above.

### Limitations

In terms of the limitations of this study, first, as a single-center retrospective study, the occurrence rate of stent underexpansion may be biased by patient selection. Large-scale prospective studies with strict inclusion and exclusion criteria and controlled follow-up may provide more useful information. Second, our study has not revealed the impact of lesion-specific CAC score on clinical outcomes in patients receiving stent implantation; therefore, we cannot conclude that pre-PCI assessment of CAC by CT can improve the prognosis. Third, CT scan cannot distinguish the depth and morphology of calcium accurately, but usually only thick or circumferential superficial calcium and CNs will lead to stent underexpansion. Fourth, the sample size of CCTA cohort was relatively small for the results to be generally accepted than the NCCT cohort. Finally, these conclusions do not apply to patients with LM lesions, ostial lesions, or CTO lesions.

## Data Availability

The datasets presented in this article are not readily available because of regulation restrictions. Requests to access the datasets should be directed to Ben He, heben241@126.com.

## References

[1] Mattesini A Di Mario C. Calcium: a predictor of interventional treatment failure across all fields of cardiovascular medicine. Int J Cardiol. (2017) 231:97–8. 10.1016/j.ijcard.2017.01.054 28096040

[2] Fujii K Carlier SG Mintz GS Yang YM Moussa I Weisz G Stent underexpansion and residual reference segment stenosis are related to stent thrombosis after sirolimus-eluting stent implantation: an intravascular ultrasound study. J Am Coll Cardiol. (2005) 45(7):995–8. 10.1016/j.jacc.2004.12.066 15808753

[3] Hong MK Mintz GS Lee CW Park DW Choi BR Park KH Intravascular ultrasound predictors of angiographic restenosis after sirolimus-eluting stent implantation. Eur Heart J. (2006) 27(11):1305–10. 10.1093/eurheartj/ehi882 16682378

[4] De Maria GL Scarsini R Banning AP. Management of calcific coronary artery lesions: is it time to change our interventional therapeutic approach? JACC Cardiovasc Interv. (2019) 12(15):1465–78. 10.1016/j.jcin.2019.03.038 31395217

[5] Zhang M Matsumura M Usui E Noguchi M Fujimura T Fall KN Intravascular ultrasound-derived calcium score to predict stent expansion in severely calcified lesions. Circ Cardiovasc Interv. (2021) 14(10):e010296. 10.1161/CIRCINTERVENTIONS.120.010296 34665658

[6] Fujino A Mintz GS Matsumura M Lee T Kim SY Hoshino M A new optical coherence tomography-based calcium scoring system to predict stent underexpansion. EuroIntervention. (2018) 13(18):2182–9. 10.4244/EIJ-D-17-00962 29400655

[7] Lawton JS Tamis-Holland JE Bangalore S Bates ER Beckie TM Bischoff JM 2021 ACC/AHA/SCAI guideline for coronary artery revascularization: executive summary: a report of the American College of Cardiology/American Heart Association joint committee on clinical practice guidelines [published correction appears in Circulation. 2022 Mar 15;145(11):e771]. Circulation. (2022) 145(3):e4–e17. 10.1161/CIR.0000000000001039 34882436

[8] Agatston AS Janowitz WR Hildner FJ Zusmer NR Viamonte M Jr Detrano R. Quantification of coronary artery calcium using ultrafast computed tomography. J Am Coll Cardiol. (1990) 15(4):827–32. 10.1016/0735-1097(90)90282-T 2407762

[9] Hecht HS. Coronary artery calcium scanning: past, present, and future. JACC Cardiovasc Imaging. (2015) 8(5):579–96. 10.1016/j.jcmg.2015.02.006 25937196

[10] Grundy SM Stone NJ Bailey AL Beam C Birtcher KK Blumenthal RS 2018 AHA/ACC/AACVPR/AAPA/ABC/ACPM/ADA/AGS/APhA/ASPC/NLA/PCNA guideline on the management of blood cholesterol: a report of the American College of Cardiology/American Heart Association task force on clinical practice guidelines [published correction appears in Circulation. 2019 Jun 18;139(25):e1182-e1186]. Circulation. (2019) 139(25):e1082–143. 10.1161/CIR.0000000000000625 30586774 PMC7403606

[11] Qian Z Anderson H Marvasty I Akram K Vazquez G Rinehart S Lesion- and vessel-specific coronary artery calcium scores are superior to whole-heart agatston and volume scores in the diagnosis of obstructive coronary artery disease. J Cardiovasc Comput Tomogr. (2010) 4(6):391–9. 10.1016/j.jcct.2010.09.001 21035423

[12] Komaki S Ishii M Ikebe S Kaichi R Mori T Marume K Association between coronary artery calcium score and stent expansion in percutaneous coronary intervention. Int J Cardiol. (2021) 334:31–6. 10.1016/j.ijcard.2021.04.021 33878373

[13] Sekimoto T Akutsu Y Hamazaki Y Sakai K Kosaki R Yokota H Regional calcified plaque score evaluated by multidetector computed tomography for predicting the addition of rotational atherectomy during percutaneous coronary intervention. J Cardiovasc Comput Tomogr. (2016) 10(3):221–8. 10.1016/j.jcct.2016.01.004 26811266

[14] Budoff MJ Nasir K Kinney GL Hokanson JE Barr RG Steiner R Coronary artery and thoracic calcium on noncontrast thoracic CT scans: comparison of ungated and gated examinations in patients from the COPD gene cohort. J Cardiovasc Comput Tomogr. (2011) 5(2):113–8. 10.1016/j.jcct.2010.11.002 21167806 PMC3075464

[15] Kim YK Sung YM Cho SH Park YN Choi HY. Reliability analysis of visual ranking of coronary artery calcification on low-dose CT of the thorax for lung cancer screening: comparison with ECG-gated calcium scoring CT. Int J Cardiovasc Imaging. (2014) 30(Suppl 2):81–7. 10.1007/s10554-014-0507-8 25084979

[16] Shin JM Kim TH Kim JY Park CH. Coronary artery calcium scoring on non-gated, non-contrast chest computed tomography (CT) using wide-detector, high-pitch and fast gantry rotation: comparison with dedicated calcium scoring CT. J Thorac Dis. (2020) 12(10):5783–93. 10.21037/jtd-20-1371 33209410 PMC7656362

[17] Zheng X Xu K Yang X Yang W Zhang W Jiang Y Association between coronary artery calcium score and in-stent restenosis after drug-eluting stent implantation. Coron Artery Dis. (2022) 33(4):284–94. 10.1097/MCA.0000000000001124 35085159

[18] Räber L Mintz GS Koskinas KC Johnson TW Holm NR Onuma Y Clinical use of intracoronary imaging. Part 1: guidance and optimization of coronary interventions. An expert consensus document of the European association of percutaneous cardiovascular interventions [published correction appears in Eur Heart J. 2019 Jan 14;40(3):308]. Eur Heart J. (2018) 39(35):3281–300. 10.1093/eurheartj/ehy285 29790954

[19] Abbara S Blanke P Maroules CD Cheezum M Choi AD Han BK SCCT Guidelines for the performance and acquisition of coronary computed tomographic angiography: a report of the society of cardiovascular computed tomography guidelines committee: endorsed by the north American society for cardiovascular imaging (NASCI). J Cardiovasc Comput Tomogr. (2016) 10(6):435–49. 10.1016/j.jcct.2016.10.002 27780758

[20] Hecht HS Cronin P Blaha MJ Budoff MJ Kazerooni EA Narula J 2016 SCCT/STR guidelines for coronary artery calcium scoring of noncontrast noncardiac chest CT scans: a report of the society of cardiovascular computed tomography and society of thoracic radiology [published correction appears in J Cardiovasc Comput Tomogr. 2017 Mar - Apr;11(2):170]. J Cardiovasc Comput Tomogr. (2017) 11(1):74–84. 10.1016/j.jcct.2016.11.003 27916431

[21] Allison MA Cheung P Criqui MH Langer RD Wright CM. Mitral and aortic annular calcification are highly associated with systemic calcified atherosclerosis. Circulation. (2006) 113(6):861–6. 10.1161/CIRCULATIONAHA.105.552844 16461818

[22] Mintz GS Nissen SE Anderson WD Bailey SR Erbel R Fitzgerald PJ American College of Cardiology Clinical expert consensus document on standards for acquisition, measurement and reporting of intravascular ultrasound studies (IVUS). A report of the American College of Cardiology task force on clinical expert consensus documents. J Am Coll Cardiol. (2001) 37(5):1478–92. 10.1016/S0735-1097(01)01175-5 11300468

[23] Mintz GS. Clinical utility of intravascular imaging and physiology in coronary artery disease. J Am Coll Cardiol. (2014) 64(2):207–22. 10.1016/j.jacc.2014.01.015 24530669

[24] Friedrich GJ Moes NY Mühlberger VA Gabl C Mikuz G Hausmann D Detection of intralesional calcium by intracoronary ultrasound depends on the histologic pattern. Am Heart J. (1994) 128(3):435–41. 10.1016/0002-8703(94)90614-9 8074002

[25] Kume T Okura H Kawamoto T Yamada R Miyamoto Y Hayashida A Assessment of the coronary calcification by optical coherence tomography. EuroIntervention. (2011) 6(6):768–72. 10.4244/EIJV6I6A130 21205603

[26] Lee T Mintz GS Matsumura M Zhang W Cao Y Usui E Prevalence, predictors, and clinical presentation of a calcified nodule as assessed by optical coherence tomography. JACC Cardiovasc Imaging. (2017) 10(8):883–91. 10.1016/j.jcmg.2017.05.013 28797410

[27] Morofuji T Kuramitsu S Shinozaki T Jinnouchi H Sonoda S Domei T Clinical impact of calcified nodule in patients with heavily calcified lesions requiring rotational atherectomy. Catheter Cardiovasc Interv. (2021) 97(1):10–9. 10.1002/ccd.28896 32259392

[28] de Ribamar Costa J Jr Mintz GS Carlier SG Fujii K Sano K Kimura M Intravascular ultrasound assessment of drug-eluting stent expansion. Am Heart J. (2007) 153(2):297–303. 10.1016/j.ahj.2006.08.026 17239693

[29] Kobayashi Y Okura H Kume T Yamada R Kobayashi Y Fukuhara K Impact of target lesion coronary calcification on stent expansion. Circ J. (2014) 78(9):2209–14. 10.1253/circj.CJ-14-0108 25017740

[30] Sugiura J Watanabe M Nobuta S Okamura A Kyodo A Nakamura T Prediction of optical coherence tomography-detected calcified nodules using coronary computed tomography angiography. Sci Rep. (2022) 12(1):22296. 10.1038/s41598-022-26599-9 36566340 PMC9789942

