# Supplementary material for: Lesion-specific coronary artery calcium score to predict stent underexpansion
Source: Front Cardiovasc Med. 2025 Feb 4;12:1524390. doi: 10.3389/fcvm.2025.1524390 (PMC11832659; doi:10.3389/fcvm.2025.1524390)
Supplement: Supplementary file 1 [file Datasheet1.docx]

**Supplemental Figure 1.** Illustration of the same lesion detected by three imaging tools in LAD (A) and RCA (B)


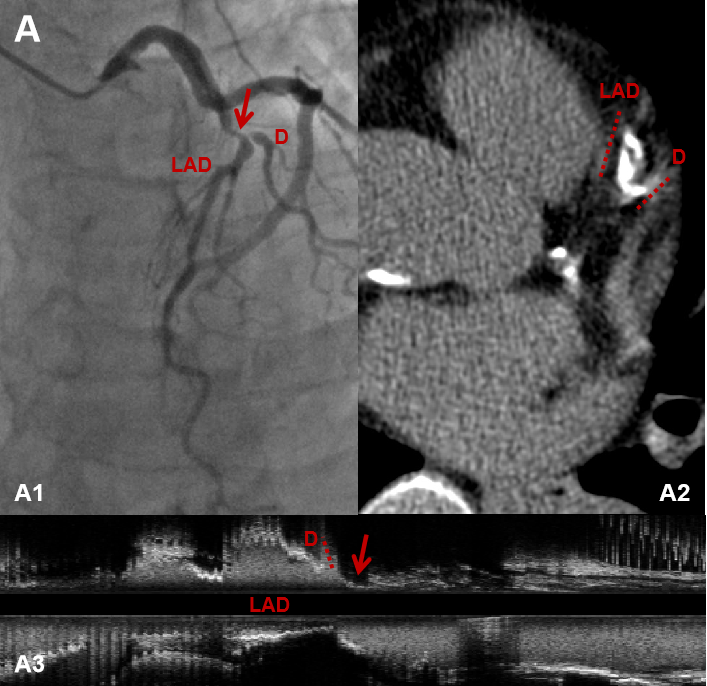

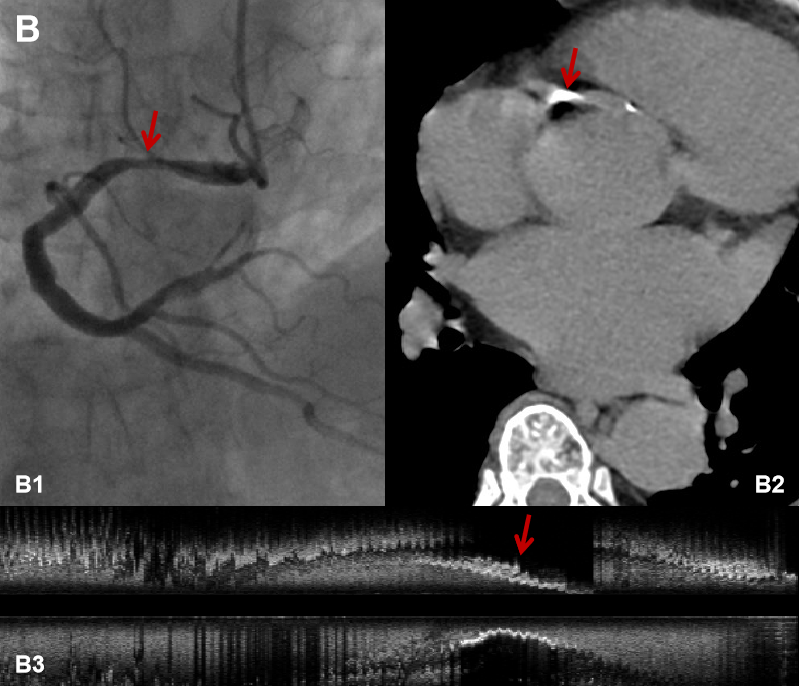


1. The red arrow indicates the same calcified lesion in LAD detected by CAG (A1), CT (A2), and IVUS (A3). The diagonal branch is an anatomical landmark. (B) The red arrow indicates the same calcified lesion in RCA detected by CAG (B1), CT (B2), and IVUS (B3). The atrioventricular sulcus is an anatomical landmark.

Abbreviation: LAD, left anterior descending artery; RCA, right coronary artery; D, diagonal branch; CAG, coronary angiography; CT, computed tomography; IVUS, intravascular ultrasound

**Supplemental Figure 2.** Distribution of IVUS-based calcium score in NCCT (A) and CCTA (B) cohort


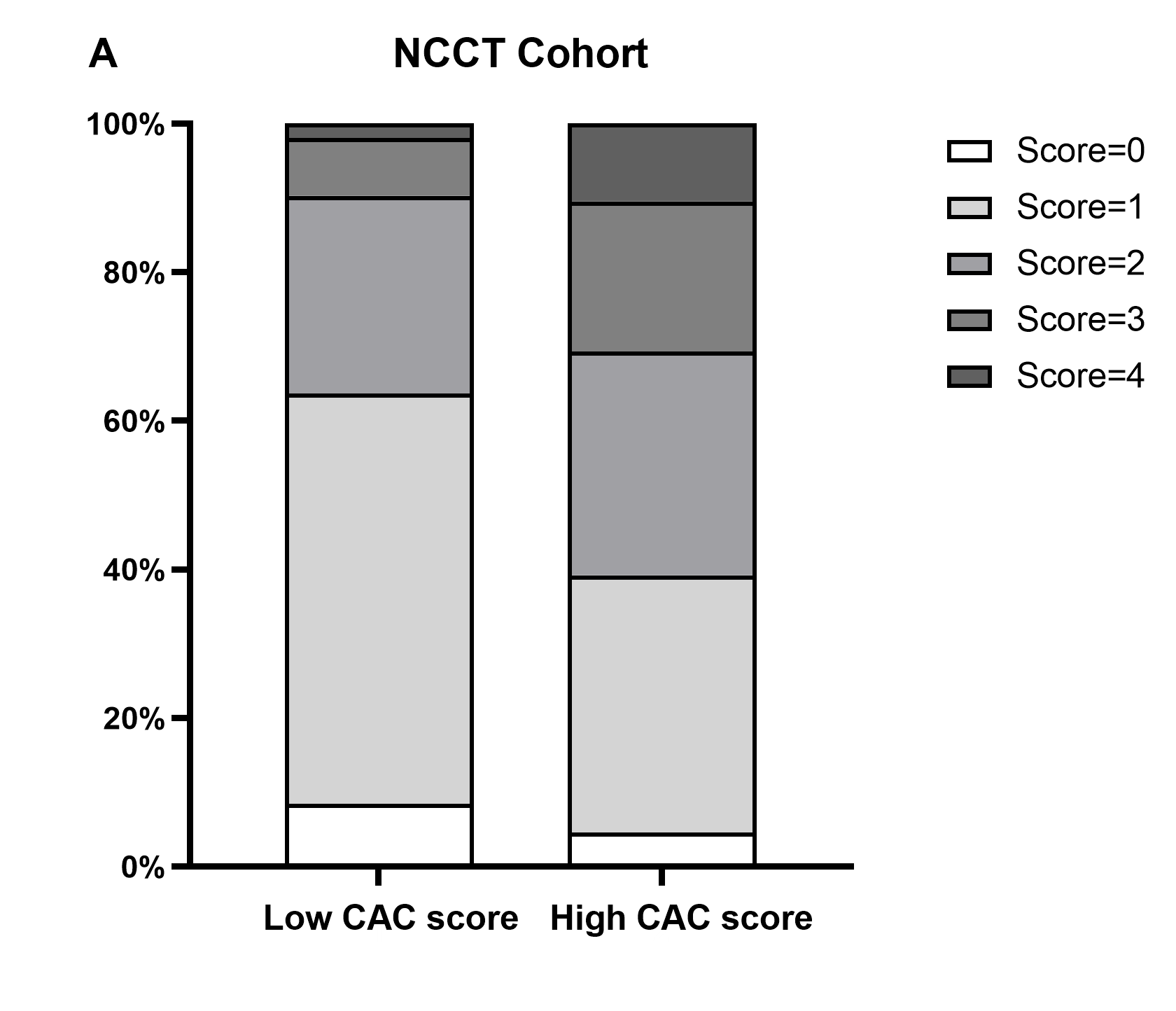

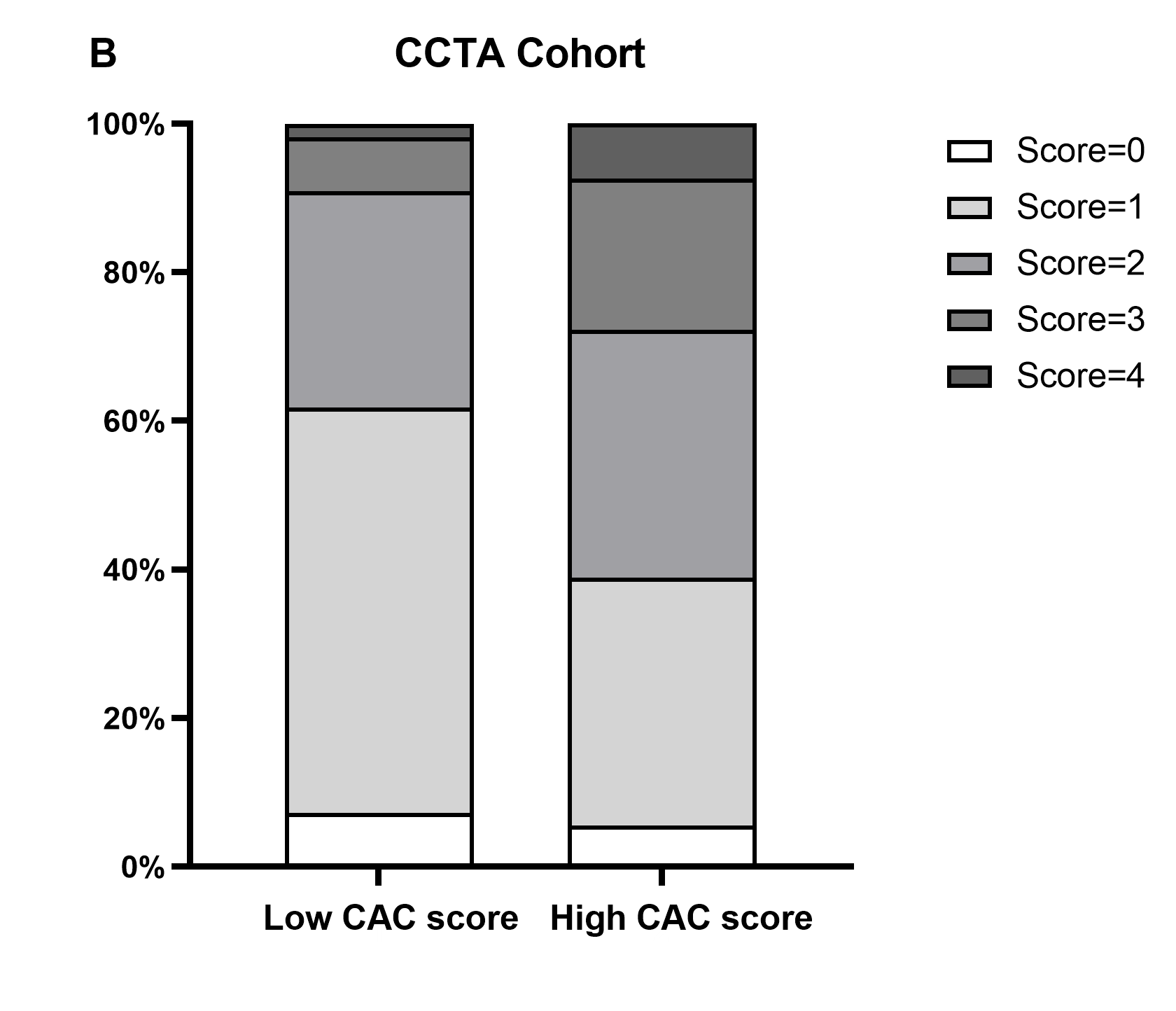


Abbreviation: IVUS, intravascular ultrasound; NCCT, non-gated non-contrast chest computed tomography; CCTA, coronary computed tomographic angiography; CAC, coronary artery calcium.
